# Atrial Natriuretic Peptide and Type 2 Diabetes Development – Biomarker and Genotype Association Study

**DOI:** 10.1371/journal.pone.0089201

**Published:** 2014-02-19

**Authors:** Amra Jujić, Peter M. Nilsson, Gunnar Engström, Bo Hedblad, Olle Melander, Martin Magnusson

**Affiliations:** 1 Department of Heart Failure and Valvular Disease, Skåne University Hospital, Malmö, Sweden; 2 Department of Clinical Sciences, Lund University, Malmö, Sweden; 3 Center of Emergency Medicine, Skåne University Hospital, Malmö, Sweden; University of Lille Nord de France, France

## Abstract

**Background:**

We have recently shown that low plasma levels of mid-regional atrial natriuretic peptide (MR-ANP) predict development of diabetes and glucose progression over time, independently of known risk factors for diabetes development. However, since MR-ANP levels might be influenced by unknown factors causing diabetes, we cannot rule out that such relationship might be confounded. Previous studies have shown an association of a single nucleotide polymorphism rs5068 on the natriuretic peptide precursor A (NPPA) locus gene with higher levels of circulating ANP. Since gene variants are inherited randomly and not subject to confounding, we aimed to investigate whether the variant rs5068 within the NPPA locus is associated with incident type 2 diabetes.

**Methods:**

We genotyped the variant rs5068 within the NPPA locus in 27,307 individuals without known diabetes from the Malmö Diet Cancer Study. Incident diabetes was retrieved through national and regional registers (median follow-up time of 14 years, 2,823 incident diabetes cases).

**Results:**

In Cox regression analysis adjusted for age, sex and BMI, we found that the carriers of at least one copy of the G allele of rs5068 had lower likelihood of incident diabetes within 14 years (HR = 0.88, 95% CI 0.78–0.99, p = 0.037).

**Conclusion:**

Our results indicate a role of the ANP system in the etiology of type 2 diabetes and might help provide insight in the metabolic actions of natriuretic peptides and the pathophysiology of type 2 diabetes.

## Introduction

The natriuretic peptides (NPs), which are secreted from cardiomyocytes in response to cardiac wall stress, play an important role in the regulation of blood pressure, intravascular volume, and cardiac remodeling. The NPs consists of atrial natriuretic peptide (ANP), brain natriuretic peptide (BNP) and C-type natriuretic peptide, where ANP and BNP are secreted by the heart in response to increased volume and pressure load [1]. Whereas the role of NP deficiency in hypertension is well established by genetic studies in both humans and animals [2]–[4] and in line with the physiological blood pressure lowering actions of the hormone, the physiological actions of NP related to glucose metabolism have just recently been accounted for [5]. Indeed, experimental data supports the notion that low ANP levels predisposes to development of diabetes and insulin resistance through an activation of the renin-angiotensin system [6]–[13], and direct beneficial effects of ANP on the beta-cell have also been reported [14], [15]. Large cross-sectional studies have shown lower levels of NP in patients with metabolic syndrome, fasting glucose and insulin resistance [16]–[18] and we recently published data showing that reduced levels of circulating midregional atrial natriuretic peptide (MR-ANP), but not the N-terminal prohormone of brain natriuretic peptide (NT-proBNP), predicted new onset diabetes mellitus (DM) as well as degree of fasting glucose progression over time (defined as the difference between fasting glucose concentration at the reexamination and at the baseline examination divided by the follow up time) at the population level, independently of diabetes risk factors and renal function during 16 years of follow-up, thus suggesting that low ANP might be causally related to diabetes development [5].

There is also evidence of a potential causal inverse association between NT-pro-BNP levels and risk of incident type 2 diabetes mellitus (T2D) in a recent large prospective cohort without T2D and cardiovascular disease [19]. Nevertheless, the risk of confounding and reverse causality in these studies cannot be easily disregarded. Interestingly, we recently published a study that showed an association of a single nucleotide polymorphism rs5068 on the natriuretic peptide precursor A (NPPA) locus gene with higher levels of circulating ANP as well as lower interindividual blood pressure (BP) [4]. The BP lowering effects of rs5068 have also been replicated in larger cohorts, where the variant was associated with 10% lower odds of hypertension [20]. Cannone and associates demonstrated that the minor allele of rs5068 is associated with favorable cardiometabolic phenotype including lower BMI and waist circumference in a general US population [34], findings that were replicated in a Mediterranean population, but surprisingly no association with T2D [39]. Another possible protective role of NPs have recently been observed in our study where the minor allele of rs5068 was associated with lower left ventricular mass and lower prevalence of left ventricular hypertrophy in a population free from T2D [38].

Since gene variants are inherited randomly and not subject to confounding we aimed to investigate whether the variant rs5068 within the NPPA locus, previously shown to be associated with ANP levels in plasma [4], also is associated with incident diabetes. The use of genetic variants should decrease the risk of confounding and reverse causality.

## Methods

### Ethics statement

A written informed consent was obtained from all subjects. All studies were approved by the ethical committee at Lund University (MKC:LU 51–90; MPP:Official records no. 85/2004: MPP-RES:LU 244-02).

### Subjects

The Malmö Diet and Cancer Study (MDC) is a prospective population-based study (n = 30,447, DNA available on n = 28,767) where baseline examinations including anthropometrical measurements and blood sample donations were performed between 1991 and 1996 [21], [22]. A complete description of the study population has been given elsewhere [23].

Prevalent diabetes at baseline was defined as self-reported physician diagnosis of T2D or use of DM medication or fasting whole blood glucose of ≥6.1 mmol/L (samples available in 5,405) [24]. After excluding patients with prevalent DM (n = 1,311) at baseline examination we were able to genotype the variant rs5068 within the NPPA locus in 27,308 subjects, and of these complete data of all covariates (age, sex and body mass index (BMI)) were available in 27,307 subjects (mean age 58 years; 60.6% women). Incident diabetes (median follow-up time of 14.0±3.8 years, 2,823 incident diabetes cases) was retrieved through national and regional registers as follows: The Malmö Hba1c register (MHR) that analyzed all Hba1c samples at the Department of Clinical Chemistry obtained in institutional and non-institutional care in Malmö are from 1988 and onwards; The Swedish National Diabetes Register (NDR) [26]; The Regional Diabetes 2000 register of the Scania region [27]; The Swedish National Patient Register covering all somatic and psychiatric hospital discharges and hospital based outpatient care [28]; Swedish Cause-of-Death register (DM as cause of death) [29] and Swedish Prescribed Drug Register (prescription of anti-diabetic medication) [30]. In addition, in subjects that provided fasting glucose samples at baseline, the diagnosis of incident T2D was obtained via oral glucose tolerance test results or fasting plasma glucose of ≥7 mmol/L or self-reported physician diagnosis or use of anti-diabetic medication in a re-examination conducted between 2007 and 2012 [25]. Furthermore, since nearly one third of the MDC participants also participated in Malmö Preventive Project [31], T2D diagnosis was obtained by identifying subjects with fasting plasma glucose ≥7 mmol/L obtained in the MPP Re-Examination Study between 2002 and 2006.

### Genotyping

The SNP was genotyped by “assay by design” TaqMan probes using a real-time polymerase chain reaction assay on an ABI 7900HT (Applied Biosystems, Foster City, CA, USA), according to the manufacturer's instructions. Twenty percent of the samples were run in duplicates. All genotypes were called by two different investigators.

Hardy Weinberg equilibrium significance analysis did not show any significant deviation (p-value = 0.20).

### Statistical analysis

Cox regression models were used to calculate hazard ratios (HR) for incident T2D at follow up in a model adjusted for age and sex, and in a model adjusted for age, sex and BMI. Due to a small number of minor homozygotes, we assumed a model (AA versus AG+GG) where carriers of one or two copies of the minor G-allele were pooled (AG+GG) because GG genotype is very rare and thus would have large and unreliable confidence intervals by itself. An additional analysis using an additive model was performed using Cox regression models and adjusted for age, sex and BMI. All analyses were performed in SPSS Windows 20.0 (SPSS Inc, Illinois, USA). A two-tailed P value<0.05 was considered statistically significant. The median (interquartile range, IQR) follow-up time was 14 years.

## Results

**Figure 1:**
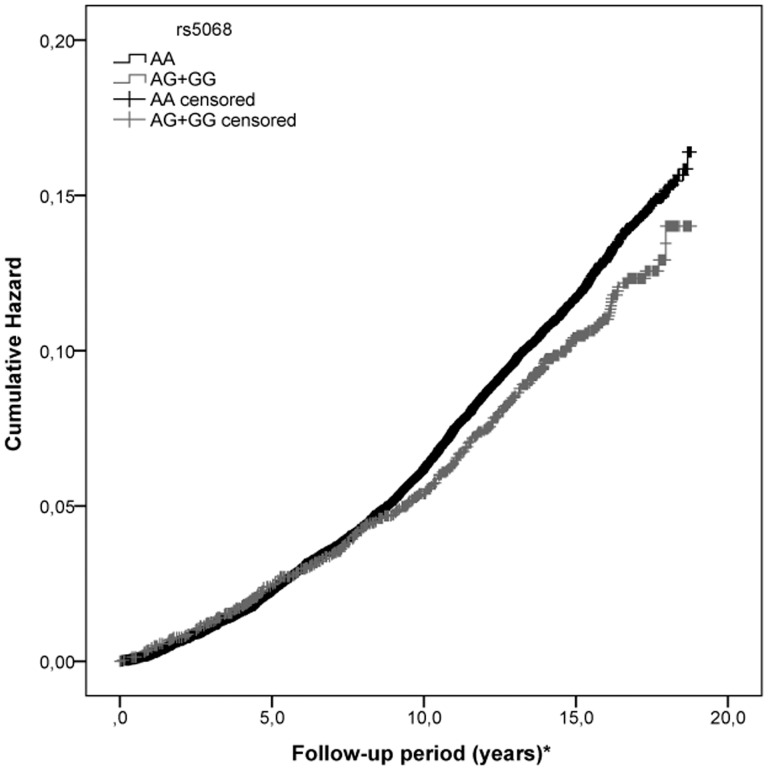
Risk of diabetes occurring in a AA versus AG+GG model of rs5068 allele. Cumulative incidence of T2D over a mean follow-up of 14 years for major allele (AA) and G allele (AG+GG) of rs5068. * Follow up period in years until first diabetes event, or, for censored cases, death, emigration or last follow up date.

Baseline characteristics of the population with and without incident T2D are listed in **Table 1**. Baseline characteristics of the population divided by each allele (AA, AG and GG) are listed in **Table 2**. Genotype frequencies for rs5068 are: AA, 88.5% (n = 24,157); AG, 11.2% (n = 3065) and GG, 0.3% (n = 85). In crude Cox regression analysis using a AA versus AG+GG model, carriers of at least one copy of the G allele of rs5068 showed lower likelihood of developing T2D within 14 years (HR = 0.88, 95% CI 0.78–0.99, p = 0.043). Results for a Cox regression analysis in a model adjusted for age and sex (HR 0.88, 95% CI 0.78–0.99, p = 0.041) and a model adjusted for age, sex and BMI (HR = 0.88, 95% CI 0.78–0.99, p = 0.037) showed significant association for the carriers of at least one copy of the G allele of rs5068 and lower likelihood of developing T2D within 14 years as presented in **Table 3**. An additional Cox regression analysis using an additive model showed significant association for the minor G allele (HR 0.89, 95%CI 0.79–0.99, p = 0.044). Proportional hazard assumption was tested using log-minus-log plots. The Kaplan Meier method was used to estimate the cumulative hazard of diabetes development in the total sample for two groups of subjects (AA and AG+GG) as presented in **Figure 1**, showing that G allele carriers (AG+GG) had lower cumulative incidence of T2D. The difference was statistically significant according to log rank test (p = 0.043), compared to subjects with the major allele (AA).

**Table 1:** Baseline Characteristics of the Study Population.

	Subjects without incident T2D	Subjects with incident T2D	Total sample
**N**	24,484	2,823	27,307
**Sex (% women)**	61.8	48.7	60.6
**Age (years)**	58.0 (±7.8)	58.7 (±7.1)	58.0 (±7.7)
**BMI**	25.5 (±3.9)	28.4 (±4.4)	25.8 (±4.0)
**Follow up time (years) (IQR1-IQR3)**	15.1 (13.9–16.7)	9.5 (5.2–12.4)	14.8 (13.5–16.4)
**AA (n (%))**	21626 (88.3)	2531 (89.7)	24157 (88.5)
**AG+GG (n (%))**	2,858 (11.7)	292 (10.3)	3,150 (11.5)

Values are displayed as means (± standard deviation) or frequency in percent (%). BMI; body mass index; IQR: median and 25–75% interquartile range. Genotype frequencies of rs5068 are presented using a dominant model; major allele represented by AA, G alleles represented by AG+GG.

**Table 2:** Baseline Characteristics of the Study Population per Each Allele.

	AA	AG	GG
**N**	24,157	3,065	85
**Sex (% women)**	60.5	61.0	62.4
**Age (years)**	58.0 (±7.7)	58.2 (±7.7)	58.0 (±7.7)
**BMI**	25.8 (±4.0)	25.7 (±3.9)	27.0 (±5.1)
**Follow up time (years) (IQR1-IQR3)**	13.9 (13.6–16.4)	13.9 (13.6–16.4)	14.8 (13.8–16.5)

Values are displayed as means (± standard deviation) or frequency in percent (%). BMI; body mass index; IQR: median and 25–75% interquartile range.

**Table 3:** Association of rs5068 and incident T2D.

	Model 1			Model 2		
	Beta (SE)	P-value	HR (95% CI)	Beta (SE)	P-value	HR (95% CI)
**Age**	0.022 (0.003)	<0.001	1.022 (1.017–1.027)	0.015 (0.003)	<0.001	1.015 (1.010–1.021)
**Sex**	−0.544 (0.038)	<0.001	0.580 (0.539–0.625)	−0.551 (0.038)	<0.001	0.576 (0.535–0.621)
**rs5068**	−0.126 (0.062)	0.041	0.881 (0.781–0.995)	−0.129 (0.062)	0.037	0.879 (0.779–0.992)
**BMI**				0.154 (0.004)	<0.001	1.166 (1.158–1.175)

Model 1: adjusted for age and sex; model 2: adjusted for age, sex and BMI.

Age: per years; sex: women vs. men; rs5068: AA vs. AG+GG. Values are hazard ratios (95% confidence intervals) for incident T2D. BMI; body mass index.

## Discussion

Several studies, including our own [5], have suggested a possible role of natriuretic peptides in etiology of diabetes, however these prospective associations might have been subject to confounding and reverse causality [5], [19], [32]. The key finding of this large prospective study is that the carriers of at least one copy of G allele of rs5068, an SNP that was previously shown to be associated with MR-ANP levels in plasma [4], had lower likelihood of incident diabetes at a follow up of ∼14 years. Since gene variants are inherited randomly and are not subject to confounding, our data suggest a causal role of the ANP metabolism in diabetes development.

The minor allele of rs5068 is associated with higher levels of circulating ANP, as well as with lower BP [4], [20]. A recent study explored associations between NP system polymorphisms and cardiovascular outcome in a general population with coronary artery disease, demonstrating associations between the rare allele of rs5068 and lower likelihood of hypertension, but also higher circulating NP levels [33]. There is also evidence of association between rs5068 and cardiometabolic protection observed by Cannone and associates [34], which suggests that this particular single nucleotide polymorphism (SNP) or genetic loci in linkage disequilibrium might have a protective role via metabolic actions of natriuretic peptides [1]. Cannone and associates managed recently to replicate these findings in a Mediterranean population where carriers of the G allele of rs5068 had lower BP, BMI, and prevalence of hypertension and metabolic syndrome, but found no correlation between rs5068 and T2D after adjusting for BMI. However, since the sample size in this study is relatively small (n = 804), it warrants replication in larger cohorts [39]. Surprisingly, in our study, the carriers of the minor allele (GG) of rs5068 had somewhat higher BMI compared to AA and AG carriers (**Table 2**), but we believe that this is due to the small number of minor allele carriers (n = 85), and this discrepancy was attenuated when using a model where AG+GG alleles were pooled into one variable (AA, 25.8 versus AG+GG, 25.7). Therefore, we do not believe that the actions of rs5068 are mediated by lower BMI.

Genetic studies in both animals and humans have established the role of ANP in hypertension [2]–[4], and there is an increasing number of studies suggesting that ANP is involved in glucose metabolism and plays a role in clustering of diabetes and hypertension. The mechanism of action of ANP is not completely understood, but experimental data suggest that low levels of ANP promote future development of insulin resistance and diabetes via activation of the renin-angiotensin system which in turn increases oxidative stress and inflammatory response, causes cross-talk between insulin and angiotensin signalling systems, and disturb glucose transporting [6]–[13]. ANP infusion has been shown to increase circulating levels of insulin in humans by 50%, and there is evidence of inhibition of glucagon secretion via direct effects on pancreatic β-cells [14], [15]. Recently, Arora and associates suggested that rs5068 acts by altering NPPA gene transcription or mRNA stability by preventing binding of miR-425 and results in higher ANP levels, and thus probably not by altering secretory mechanisms or peptide clearance [40]. Since ANP and BNP hormone levels are highly correlated and share the same receptors for mediating physiological effects [4], [32], there is an on-going discussion whether the association of natriuretic peptides and T2D is mainly mediated by effects of the ANP or the BNP system. The minor allele of rs5068 have, like several other SNPs involved in regulating natriuretic peptide levels, shown an association with ANP and BNP levels, but the association between rs5068 and ANP levels is almost 3-fold stronger than between the minor allele and BNP levels [4]. One possible explanation to this could be sub-clinical left ventricular systolic and/or diastolic dysfunction leading to relatively more BNP as compared to ANP secretion thus attenuating the relationships between N-BNP and incident diabetes [35]. ANP is namely considered to be mainly secreted by the cardiac atria, thus being less sensitive to increases in intra-ventricular pressure/hemodynamic stress than BNP, which in turn is mainly secreted by the cardiac ventricles [1], [36]. Accordingly, N-BNP has also proven to be a more sensitive marker for the diagnosis of mild forms of left ventricular dysfunction compared to N-ANP [37]. Despite the linkage disequilibrium between large parts of the NPPA and NPPB genes, the rs5068 does in fact primarily affect ANP levels which is supported by stronger rs5068 association with ANP than with BNP [4]. The linkage disequilibrium between the two loci and the correlation between ANP and BNP (driven by similar stimuli of secretion of the two hormones, e.g. cardiac wall stress) may cause a “non-functional” relationship between rs5068 and BNP. Taking all this in perspective, we cannot eliminate the possibility that the actions of BNP also contribute to the observed association with T2D. Also, effect sizes for most SNP's are small, thus explaining only a very small proportion of the expected heritability. Understanding complex traits and variation in humans will probably remain a challenge. Since T2D is a multifactorial disease with a range of known risk factors contributing to its pathogenesis, those risk factors should be taken into account when conclusions are drawn about associations. In this study of inhabitants of Malmö, Sweden, we tried to adjust for risk factors which we had access to (age, gender, BMI). However, we cannot rule out that other, known and unknown risk factors, would have affected the outcome of our analysis. Also, any association between a SNP of interest and health outcome may be due to linkage disequilibrium between the different SNP's influencing disease risk. The total MDC cohort is female biased (60.6% women); however, the gender distribution in the population with incident T2D is more balanced (48.7% women). This could be explained by the fact that T2D is equally prevalent among women and men in most populations, and is in line with some evidence of male preponderance in subjects in early middle age [41]. As this is a longitudinal observational study, it shares the inherent limitations about causality and control as all observational studies. Still, the genetic effect is not expected to be hampered by such confounding, as the genome is constant throughout life. Also, our data was collected at a single regional centre which limits the applicability to other populations and allows the influence of community characteristics.

## Conclusion

Our results offer an indication for a role of the ANP system in the etiology of type 2 diabetes and might help provide insight in the metabolic actions of natriuretic peptides and the pathophysiology of T2D.
